# Setting competencies and standards for a European Leadership Program in Geriatric Medicine: “The European Academy for Medicine of Ageing (EAMA) reloaded”

**DOI:** 10.1007/s41999-018-0052-4

**Published:** 2018-05-08

**Authors:** Regina Elisabeth Roller-Wirnsberger, Nele van den Noortgate, Sylvie Bonin-Guillaume, Karen Andersen-Ranberg, Anette Hylen Ranhoff, Thomas Münzer, Tomasz Grodzicki, Simon Conroy, Francesco Landi, Louis Mieiro, Ulrike Dapp, Robertus van Deelen, Rannveig Sakshaug Eldholm, Nicolas Martinez-Velilla, Katrin Singler

**Affiliations:** 1 Board of the European Academy for Medicine of Ageing (EAMA), Ghent, Belgium; 2 Department of Internal Medicine, Medical University of Graz, Auenbruggerplatz 15, 8036 Graz, Austria; 3 Department of Geriatric Medicine, Ghent University, Ghent, Belgium; 4 Department of Geriatric Medicine, Aix Marseille University, Marseille, France; 5 Department of Geriatrics, Odense University Hospital, Odense, Denmark; 6 University of Bergen, Bergen, Norway; 7 Geriatrische Klinik, St. Gallen, Switzerland; 8 Department of Geriatrics, University of Zürich, Zürich, Switzerland; 9 Department of Internal Medicine and Geriatrics, Jagiellonian University Medical College, Kraków, Poland; 10 Department of Health Science, University of Leicester, Leicester, UK; 11 Department of Geriatrics and Rehabilitation, Catholic University of Sacred Heart, Rome, Italy; 12 MRC Unit for Lifelong Health and Ageing, University College London, London, UK; 13 Department of Geriatrics, Albertinen-Haus Hamburg, Hamburg, Germany; 14 Scientific Department, University of Hamburg, Hamburg, Germany; 15 Department of Geriatrics, Spaarne Gasthuis, Haarlem, The Netherlands; 16 Department of Neuromedicine and Movement Science, Norwegian University of Science and Technology (NTNU), Trondheim, Norway; 17 Servicio de Geriatría, Complejo Hospitalario de Navarra, Pamplona, Spain; 18 Institute of Biomedicine of Ageing, Friedrich Alexander University Erlangen-Nürnberg, Erlangen, Germany; 19 Department of Geriatrics, Klinikum Nürnberg, Paracelsus Private Medical University, Nuremberg, Germany

**Keywords:** European Academy for Medicine of Ageing, Learning objectives, Leadership programme, Competence based programme, Kirkpatrick evaluation

## Abstract

**Background:**

The European Academy for Medicine of Ageing (EAMA) was founded in 1995 as an “Advanced Postgraduate Course in Geriatric Medicine”, in order to train future key opinion leaders in geriatric medicine. Recent changes across European Healthcare systems have changed the needs for leadership competences for geriatricians. Therefore, it became mandatory to further develop EAMA’s learning objectives catalogue.

**Materials and methods:**

Following a comprehensive needs assessment among students and visiting professors of the EAMA, a template containing seven key domains derived from the needs assessment was developed. EAMA professors had the chance to feedback learning objectives aligned with the seven domains. Feedbacks were transcribed into a first draft of a learning objectives catalogue during this meeting. This first draft was reflected with EAMA network members (former EAMA students) and finalized following a second focus group among board members.

**Results:**

24 learning objectives which cover the spectrum of knowledge, skills and attitudes necessary to develop leadership roles in geriatric medicine are included in the new EAMA learning objectives catalogue. Rate of agreement achieved in open ratings was > 90% for all selected items among the board members.

**Conclusions:**

The recently developed learning objectives catalogue of EAMA presented within this publication reflects a clear shift from knowledge-based education and training towards a comprehensive programme design for leadership development.

## Background

The concept of geriatric medicine has been proven effective: nowadays, the Comprehensive Geriatric Assessment represents the gold standard for effective care of frail older people, who are prone to functional decline [1]. However, postgraduate training in geriatric medicine varies across Europe, and consistent undergraduate teaching is still uncommon in this field [2]. Therefore, the demand to train trainers and leaders in the field to support professional development in all countries of Europe is strong.

The European Academy for Medicine of Ageing (EAMA) was founded in 1992 as an Advanced Postgraduate Course in Geriatric Medicine and addresses the demand for leadership in geriatric medicine across Europe [3]. Most of the professors teaching in EAMA are former EAMA students. Selections of students follow structured criteria of workforce experience and academic background. Since its inception the major objective has been to improve knowledge and skills in geriatric medicine for emerging leaders in geriatrics and to establish a network among those doctors responsible for the care of older people across Europe [4]. The 2 years course (provided as four 1-week life educational events) has outlined clear learning objectives since then. Teaching methods included traditional class room teaching and learning sets using interactive discussions and presentations, also given by students. Both the teachers’ and students’ activities were evaluated by students.

Given the changes in public health due to ageing across Europe, the demand for progressive development of the established EAMA program became evident [5]. The current publication describes the process and results of the development of a new learning objectives catalogue (LOC) for attendees of EAMA.

## Methods

### Needs assessment

To determine the needs for new learning objectives, authors chose a multidimensional approach.

A general needs assessment for leadership knowledge, skills and attitudes was performed using feedback evaluation aligned with Kirkpatrick’ evaluation criteria [6] of recent courses.

In addition, informal and non-structured interviews with expert teachers during their stay in EAMA, reflecting the current goals and learning objectives were performed. Content was elaborated and extracted and, if possible, translated into overarching domains.

**Fig. 1:**
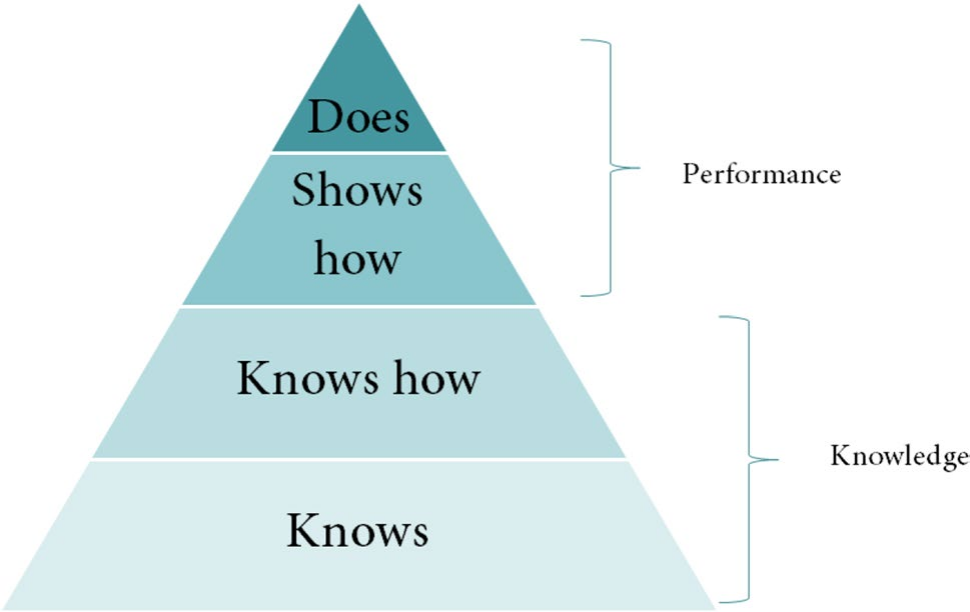
The concept of Miller’s pyramid of competences and how the level of learning objectives is aligned with educational levels [9]

Additionally, a structured focus group with participation of all EAMA board members was held. Like in the interviews with expert teachers before, all domains to be covered due to board members’ opinion were collected. Domains were than clustered according to competence levels [7]. The objectives detected were than transcribed into a template of learning objectives structured to Bloom’s taxonomy [8] and transferrable to Miller’s pyramid of competence [9] (see Fig. 1).

Using this concept, it was possible to align learning content with needs expressed by EAMA students.

The first draft of a learning objectives catalogue was than presented to board members of EAMA for further evaluation.

### Focus group of EAMA board members

The first draft of learning objectives created upon student’s feedbacks and on written programme evaluation feedbacks from 2014 to 2016 and interviews is shown in Table 1.

**Table 1:** The first draft of domains possibly to be covered in a new EAMA learning objectives catalogue. The domains were developed according to the results obtained from an open needs assessment among EAMA students and visiting professors and built upon EAMA evaluation feedbacks from courses held from 2014 to 2016

Domain
Function as medical experts, integrating all roles of an expert to provide optimal, ethical and patient-centred medical care (e.g. basic and clinical research, profound competencies on knowledge and skills in all fields of geriatric medicine)
Interpersonal and communication skills (e.g. presentation techniques, etc.)
Development of geriatric services/administrative duties (e.g. project management, training on leadership, quality control, etc.)
Interdisciplinary team management (including communication skills, etc.)
Creation, dissemination, application and translation of medical knowledge (including educational skills, etc.)
Advocacy of well-being of individual patients, communities, and populations (including advocacy of the specialisation of geriatric medicine, etc.)
Self-management, self-resilience

The draft included seven domains aligned with the CanMedRoles [7]. EAMA Board members were given the opportunity to reflect upon these domains and to introduce possible learning objectives according to their individual experience. In a next step, the two coordinating board members (RRW, KS) transcribed the raw data into a learning catalogue format aligned with Miller’s pyramid of competence [10] (see Fig. 1) and used semantic background aligned with Bloom’s taxonomy [8]. The taxonomy describes a set of three hierarchical models used to classify educational learning objectives into levels of complexity and specificity. The three lists primary developed by Benjamin Bloom cover the learning objectives in cognitive, affective and sensory domains. The cognitive domain has mainly been used to structure curriculum learning objectives in the past [8].

The first “raw version” of learning objectives was summarised by the two coordinating board members. All 10 board members were then invited to rate the new learning objectives catalogue in an open focus group format during one of the EAMA courses.

### Student’s feedback on preliminary draft of LOS catalogue

The preliminary version of the new learning objectives catalogue was sent to five randomly assigned former EAMA students (EAMA network members at advanced stage of academic career and geographically distributed across the European Union) asking for feedback on the following parameters: relevance for further career planning, options for migration within the European Union, need for rephrasing of learning objectives (LOs) for better understanding.

Feedback was evaluated by the EAMA board members independently, rated and transcribed and introduced in the final draft of the learning objectives catalogue afterwards.

### Final focus group of experts

The final consensus building for LOs catalogue presented in this paper was achieved by a teleconference call held on February 2017 among board members of EAMA. All board members had been sent out the final proposal for the LOs catalogue including former students’ comments and board members’ reflections upon. All comments were discussed and evaluated towards current training standards of the EAMA course and transferability to the future course outline of EAMA as planned by the board.

## Results

### Results from needs assessment

The biggest strength of the EAMA programme outlined by students was the network building. However, many students expressed their wish to change venue for the meetings. Students felt that gain of knowledge was high. However, the existing learning style did not seem to support their personal learning styles. Furthermore, the wish for extended training for leadership skills became obvious from the students’ feedback.

Results from interviews with visiting professors in EAMA did not have high impact on the development of the primary LOs list. Visiting professors reflected upon EAMA with its long standing tradition, however, could not contribute to the whole programme development as their visits usually did not last longer than 2 days of a programme week.

### Results from focus groups with EAMA board members

In a first raw version of the new learning objectives catalogue, each of the seven domains (see Table 1) included detailed learning objectives created and suggested by board members. In total professors developed eleven up to 28 new learning objectives per domain (data not shown).

The transcription of the LOs consented by the whole EAMA board finally contained overall 24 items which cover the spectrum of knowledge, skills and attitudes necessary to develop leadership roles in geriatric medicine. Rate of agreement achieved in open ratings was > 90% for all selected items among the board members.

Knowledge domains of the new EAMA curriculum mainly cover topics related to professional work of geriatricians, critically reflecting daily business towards evidence, but also considering strength of bottom-up approaches of best practice models or eminence delivered by experts in the field (see Table 2).

**Table 2:** Learning objectives for a full 2-year course of the European Academy of Medicine of Ageing (EAMA)

Knowledge *EAMA graduates will be able to…* … plan care based on competent critical appraisal of evidence on micro (individual patient), meso (service) and macro (societal, public health) levels … synthesize an approach drawing upon the evidence base and adapting to complex patients … develop and apply a bio-psycho-social approach to relevant geriatric and gerontological topics informed by key international leaders … understand and translate basic or clinical research as an expert in geriatric medicine … compare and contrast the general structure of geriatric care from different European perspectives
Skills Communication *EAMA graduates will be able to…* … review and select different established and emerging communication techniques, in order to improve communication with the target audience … interact effectively in different roles such as participant, chair or moderator in group discussions, giving feedback, etc. … successfully impart topics of geriatric medicine to different audiences, e.g. lay people, politicians, hospital administrators, allied health care professionals and colleagues in other disciplines to promote geriatric medicine …reflect the influence of sociocultural differences in communication and interaction and adapt own strategies accordingly …recognise, prevent and manage conflicts and crises (patient/family, colleagues, administrators …)
Skills Leadership/leadership competence at microlevel *EAMA graduates will be able to…* …analyse personal strengths to be integrated into individuals’ leadership profiles …develop leadership competences in geriatric medicine including coaching, delegating, team building, etc. … analyse personal profile and strength within the interdisciplinary team … critically reflect upon personal career development, plans and strategies and to identify specific objectives leading towards a career plan for him/herself as well as mentees
Skills Leadership competence at mesolevel *EAMA graduates will be able to…* … synthesize various inter- and multi-professional aspects of a geriatric team towards a common therapeutic plan of care reflecting patient-centred outcomes … coach change management in geriatric medicine based on evidence, lead and broaden implementation processes to scale up .. develop new geriatric services or adapt geriatric services to new situations, considering both, scientific advances and local requirements and needs … improve quality in daily practice by critical use of elements of quality management, e.g. development of a quality improvement (PDSA) cycle, etc.
Skills Leadership competence at macrolevel *EAMA graduates will be able to…* …establish and maintain active networks across Europe and the rest of the world
Skills Research *EAMA graduates will be able to…* … identify clinical problems, critically appraise evidence for solutions and generate new research questions (innovation cycle) … exploit research questions into sustainable networks and funding structures (including project management, formation of consortia and others)
Skills Education and teaching *EAMA graduates will be able to…* … create and adapt learning objectives and teaching strategies to target groups and in order to transfer knowledge and understanding … improve personal teaching competences by critical analysis of board members’ performances, expert and student speakers … create a network to European organisations linked with geriatric medicine and care in order to contribute to trans-national teaching or research activities … choose and develop learning action sets and teaching strategies (communication, considering communication towards target groups)
Skills Advocacy *EAMA graduates will be able to…* … demonstrate behaviours as an ambassador of geriatric medicine and the needs of older people locally and globally (e.g. influence decision makers, create lobbying partnership with EU/WHO) … critically appraise the policy of establishments and influence those by using tools acquired in EAMA
Attitudes *EAMA graduates will be able to…* …develop a reflective, ethically reasoned attitude on health and life in old age and implement it into daily practice, research and teaching activities … develop a personal behaviour to positively contribute to social innovation, e.g. care giver and patient empowerment … develop coping strategies towards criticism and negative impact. … critically appraise personal activity levels and potential as well as to modulate daily business efficiently (improvement of work-life balance, time management, etc.) to maintain the utmost personal resilience

Skills to be developed during the course were structured according to CanMed Roles [7], an educational framework developed by the Canadian Royal College in the 1990s. It describes the abilities that physicians require to effectively meet the health care needs of the people they serve. The framework is organized into seven thematic groups of competencies, which are expressed as CanMed roles.

The four domains developed for attitudes to be achieved during the EAMA courses were strengthened by adding leadership competences (including service development) to develop geriatric medicine at the national and international level and to contribute to innovation and research.

### Results from EAMA network feedbacks

Feedback from EAMA network members did not reveal any deletion or addition of new items in the list. Two students commented on the wording of communication and management competences learning objectives. One student requested to introduce “cultural differences in Europe” to be included in the wording of any of the learning objectives. A second comment pointed towards lobbying for partnerships between the EAMA network members.

### Results from final focus group of the EAMA board

The board discussed openly and decided not to include the two comments in the final version of the learning objectives catalogue as board members had the feeling the suggestions were very specific and covered by the final version of the catalogue anyway.

## Discussion

The European Academy for Medicine of Ageing has a long standing tradition in training of future key opinion leaders in the field of geriatric medicine. Programme evaluation has been the key ever since EAMA has been established. Due to evaluation reports of the programme as well as changing demands for geriatricians in the light of changes in European health care systems, the advancement of the EAMA curriculum became mandatory.

**Fig. 2:**
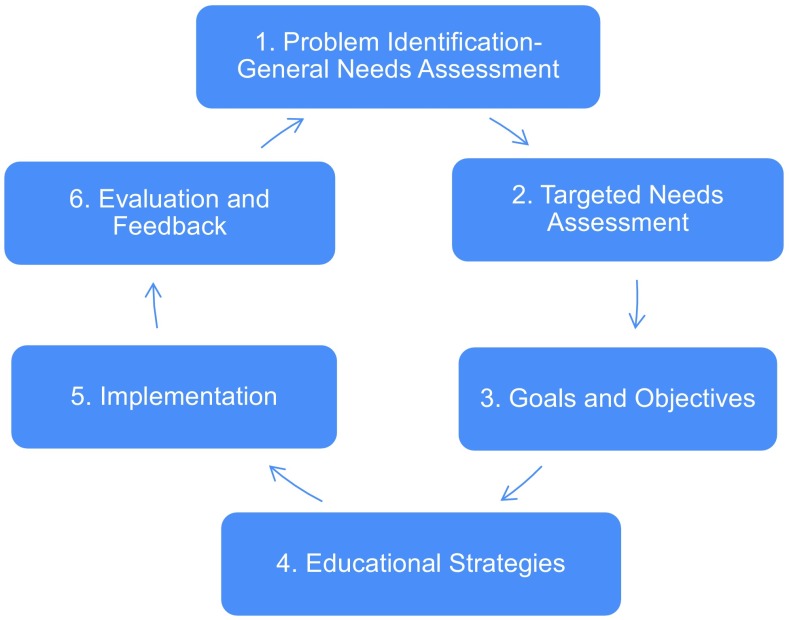
Kern’s six step approach for curricular development [10]. Each general needs assessment for development is followed by a detailed needs analysis to develop targeted programme goals and objectives. Learning environment and methodology have to be tailored according to the competence-based learning objectives developed within the Kern cycle and implementation of changes has to be monitored by evaluation closely to ensure a goal oriented programme design

Therefore, EAMA has gone through substantial curricular changes over the past 5 years. The current paper describes the development of the latest version of the EAMA learning objectives catalogue. All steps described in this publication have been planned according to Kern’s six step approach [10] (see Fig. 2).

Students and teachers were involved into the needs assessment process in an inclusive fashion and over a 12-month period, building a comprehensive mind map of all semantic and unstructured feedbacks collected during the EAMA courses.

The board chose to use an open focus group (expert panel) approach to develop a core set of learning objectives to be covered during the 4-week courses. Doing so, the board finally agreed upon an LOs catalogue, which is outlined in Table 2. As may be seen from the table, the majority of LOs is attributed to skills. Communication, leadership competencies at all levels of the public health care system for older people are the main domains covered by the new LOs catalogue of EAMA. These skills shall be carried by a set of attitudes supporting innovation and research and advocacy for older people. Only few LOs encounter knowledge and the skill to critically appraise research, innovation and evidence gathered in the care of older patients.

One major strength of the data presented here is the high internal consistency of feedbacks during the focus groups among the board members (higher than 90% for all items). Comments by former students underlined the need for a strong focus on leadership skills as outlined. Therefore, the core content of the EAMA programme currently seems to address the needs of young expert geriatricians in daily work and competition within various health care systems across Europe and also abroad. The new LOs Catalogue is, therefore, in line with WHO recommendations on workforce development published in 2013 [11].

An additional strength of our approach is the alignment with other recent educational developments in the field of geriatric medicine such as European undergraduate curriculum for geriatric competences in undergraduate medical education [12]. 7/10 EAMA board members are also members of the core group development of the undergraduate and/or postgraduate curriculum in geriatric medicine. It may be seen from the objectives developed for undergraduate students that they clearly differ in terms of competence and domains covered [12]. Similarly, recommendations for core competences in postgraduate training of future geriatricians in Europe are under development by the European Union Geriatric Medicine Society (EuGMS), Union European Medicine Societe-Geriatric Medicine Section (UEMS-GMS), European Academy for Medicine of Ageing (EAMA) and the International Association of Geriatrics and Gerontology European Region (IAGG-EUR). This joint effort of geriatricians from many European Member States tries to align content with undergraduate as well as leadership programmes, designing an integrated spiral curriculum for career development in geriatric medicine as first described by Jerome Bruner in 1960 and developed by colleges over the past decades [13, 14].

Using this “harmonising approach” it is to be expected that a well-defined programme for geriatric education fostering entrepreneurship in geriatric training will be available by the end of 2018 (http://ec.europa.eu/education/policy/strategic-framework/entrepreneurship_en). This development of a comprehensive career model in geriatric medicine will strengthen geriatric training across Europe [11].

The recently developed LOs catalogue of EAMA presented within this publication reflects a clear shift from knowledge-based education and training towards a comprehensive programme design for leadership development. This may be seen as another strength of this work. As a consequence, EAMA has changed its teaching methods towards a more and more interactive and self-reflecting teaching environment with active students’ involvement. Students are given tasks to be solved in groups with predefined roles within the groups. This concept aims to strengthen the learners’ self-concept (“I am responsible for my own decisions, however, contributing to the group’s success”). Changing environments unexpectedly during tasks aims to improve students’ readiness to learn and to provide them with necessary responsibility to adapt to changing situations.

The role of the teachers and board members in the EAMA more and more shifts towards a coaching position, providing mentorship and improving motivation and orientation during the learning experience. This approach has been proven effective and sustainable in adult education elsewhere in the literature [15]. As programme evaluation reports from students in EAMA show (data not shown in this publication) the new model of teaching is well perceived and EAMA trainees feel a clear benefit from EAMA for their professional lives at their home working places [16].

One of the major drawbacks of the work described is the small group of board members involved in the content development. At first sight, the LOs catalogue outlined in this paper was built on the experience and view of only 10 EAMA board members currently running the EAMA programme. However, to address this shortcoming two steps were implemented during the process as outlined in the methods section: focus groups of board members with needs assessment derived from students’ feedback and evaluation reports and professors’ interviews. This led to the inclusion of reflections received from end users as well as experts on top of their career in the field of geriatric medicine.

Furthermore, goal orientation of the new objectives was the key for the EAMA board, following the six-step approach by Kern [10]. The high degree of consensus among EAMA board members during the focus groups (internal consistency) and final feedbacks from EAMA network members also reflect the comprehensiveness of the process as a whole.

The new EAMA learning objectives catalogue has implications for practice during the courses. Learners’ experiences within the EAMA course differ in their level of expertise, individual constraints and preferences when starting the programme. It is, therefore, EAMA’s task to provide an environment and the resources in which each learner can develop according to their own learning style, pace and level of experience [15]. The EAMA board members have, therefore, started to cluster learning objectives for the training weeks and to design the programme accordingly. Teaching methods introduced to the programme have shifted towards a proactive role of students and putting the teachers in a role of coaches. Going through the literature, it becomes clear that the capability of faculty members as role models has an impact on the success and sustainability of continuous educational programs teaching content on professionalism. A role model faculty member is additionally effective in transferring hidden curriculum that includes all activities and teachings that happen outside the formal curriculum and is learnt through behaviours and attitudes of faculty members [16].

The development of the new EAMA learning objectives catalogue and introducing elements of modern adult education theories in the recent development poses the European Academy of Medicine of Ageing upfront all postgraduate life educational programmes currently provided. Training future key opinion leaders in the field of geriatric medicine brings EAMA into focus of future educational developments. Especially, the need for high stakes multi-professional education will be in the focus of EAMA. Reaching out the programme towards changes and needs of health care systems, to better scope with the needs of the older population in the context of multi-disciplinary services, will be one of the future directions to go. The current EAMA educational programme, built on the new learning objectives catalogue and training environment, is the core to face these future challenges.
